# *N*-(3-Chloro­phen­yl)-3-methyl­benzamide hemihydrate

**DOI:** 10.1107/S1600536810010354

**Published:** 2010-03-31

**Authors:** Vinola Zeena Rodrigues, Miroslav Tokarčík, B. Thimme Gowda, Jozef Kožíšek

**Affiliations:** a Department of Chemistry, Mangalore University, Mangalagangotri 574 199, Mangalore, India; b Faculty of Chemical and Food Technology, Slovak Technical University, Radlinského 9, SK-812 37 Bratislava, Slovak Republic

## Abstract

In the title compound, C_14_H_12_ClNO·0.5H_2_O, the N—H bond is in an *anti* conformation to the C=O bond. The two aromatic rings make a dihedral angle of 49.5 (1)°. The water mol­ecule lies on a twofold rotation axis. In the crystal, inter­molecular N—H⋯O and O—H⋯O hydrogen bonds connect the mol­ecules into a three-dimensional network.

## Related literature

For the preparation of the title compound and related structures, see: Gowda *et al.* (2008**a* ▶,b* ▶); Bowes *et al.* (2003 ▶); Rodrigues *et al.* (2010 ▶).
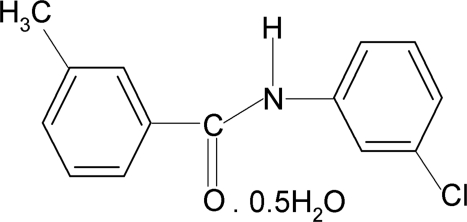


## Experimental

### 

#### Crystal data

C_14_H_12_ClNO·0.5H_2_O
*M* *_r_* = 254.71
Monoclinic, 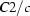

*a* = 7.6497 (3) Å
*b* = 12.6829 (5) Å
*c* = 25.9694 (10) Å
β = 95.365 (3)°
*V* = 2508.54 (16) Å^3^
*Z* = 8
Mo *K*α radiation
μ = 0.29 mm^−1^
*T* = 295 K
0.52 × 0.16 × 0.06 mm

#### Data collection

Oxford Diffraction Xcalibur diffractometer with a Ruby Gemini detector
Absorption correction: analytical (*CrysAlis PRO*; Oxford Diffraction, 2009 ▶) *T*_min_ = 0.860, *T*_max_ = 0.984
19774 measured reflections
2277 independent reflections
1894 reflections with *I* > 2σ(*I*)
*R*_int_ = 0.029

#### Refinement

*R*[*F* ^2^ > 2σ(*F* ^2^)] = 0.041
*wR*(*F* ^2^) = 0.095
*S* = 1.07
2277 reflections
164 parameters
2 restraints
H atoms treated by a mixture of independent and constrained refinement
Δρ_max_ = 0.16 e Å^−3^
Δρ_min_ = −0.20 e Å^−3^

### 

Data collection: *CrysAlis PRO* (Oxford Diffraction, 2009 ▶); cell refinement: *CrysAlis PRO*; data reduction: *CrysAlis PRO*; program(s) used to solve structure: *SHELXS97* (Sheldrick, 2008 ▶); program(s) used to refine structure: *SHELXL97* (Sheldrick, 2008 ▶); molecular graphics: *ORTEP-3* (Farrugia, 1997 ▶) and *DIAMOND* (Brandenburg, 2002 ▶); software used to prepare material for publication: *SHELXL97*, *PLATON* (Spek, 2009 ▶) and *WinGX* (Farrugia, 1999 ▶).

## Supplementary Material

Crystal structure: contains datablocks I, global. DOI: 10.1107/S1600536810010354/bt5221sup1.cif

Structure factors: contains datablocks I. DOI: 10.1107/S1600536810010354/bt5221Isup2.hkl

Additional supplementary materials:  crystallographic information; 3D view; checkCIF report

## Figures and Tables

**Table 1:** Hydrogen-bond geometry (Å, °)

*D*—H⋯*A*	*D*—H	H⋯*A*	*D*⋯*A*	*D*—H⋯*A*
N1—H1*N*⋯O2*W*^i^	0.86	2.2	3.0155 (19)	158
O2*W*—H2*W*⋯O1	0.813 (18)	1.991 (18)	2.7984 (17)	171.9 (19)

Symmetry code: (i) 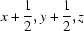.

**Fig. 1.**
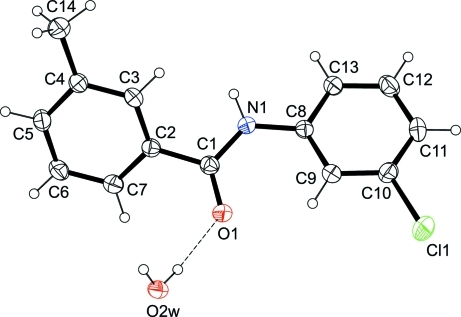
Molecular structure of the title compound showing the atom labelling scheme. Displacement ellipsoids are drawn at the 30% probability level and H atoms are represented as small spheres of arbitrary radii. Only one set of the disordered methyl H atoms is shown.

**Fig. 2.**
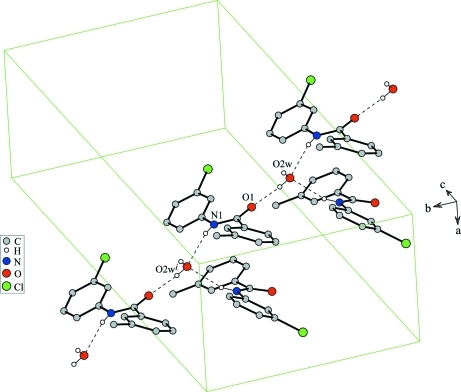
Part of the crystal structure of the title compound. Hydrogen bonds are shown as dashed lines. H atoms not involved in hydrogen bonding were omitted. Symmetry code: (i) x+1/2, y+1/2, z.

## supplementary crystallographic information

### Comment

In the present work, as part of a study of the substituent effects on the crystal structures of benzanilides (Gowda *et al.*, 2008*a,b*, Rodrigues *et al.*, 2010), the structure of *N*-(3-chlorophenyl)3-methylbenzamide hydrate (I) has been determined. In the structure, the conformations of the N—H and C=O bonds are *anti* to each other (Fig. 1), similar to those observed in *N*-(3-chlorophenyl)3-chlorobenzamide (II), *N*-(3-chlorophenyl)benzamide (III)(Gowda *et al.*, 2008*b*), 3-methyl-*N*-(phenyl)benzamide (IV)(Gowda *et al.*, 2008*a*), *N*-(2-chlorophenyl)3-methylbenzamide (V)(Rodrigues *et al.*, 2010) and the parent benzanilide (Bowes *et al.*, 2003). Further, the conformation of the C=O bond in (I) is also *anti* to the *meta*- methyl substituent in the benzoyl ring and that of the N—H bond is *anti* to the *meta*-Cl group in the aniline ring, compared to the *syn* conformation observed between the N—H bond and the *ortho*-Cl group in the aniline ring of (V).

The two aromatic rings make a dihedral angle of 49.5 (1)°. The central amide group –NH–C(=O)– is twisted by 35.1 (1)° and 15.9 (1)° out of the planes of the 3-methylphenyl and 3-chlorophenyl ring, respectively. The molecular structure is stabilized by the C9–H9···O1 intramolecular hydrogen bond (Table 1). In the crystal, the water molecule lies on a twofold rotation axis.

Intermolecular N–H···O and O–H···O hydrogen bonds connect the molecules into a three-dimensional network  (Fig.2). The water O2w oxygen lies on a twofold rotation axis 0,*y*,1/4 and its hydrogen atoms are related by the symmetry -*x*, *y*, 1/2 - *z*.

### Experimental

The title compound was prepared according to the literature method (Gowda *et al.*, 2008*a,b*). The purity of the compound was checked by determining its melting point. It was characterized by recording its infrared and NMR spectra. Single crystals of the title compound used in X-ray diffraction studies were obtained from a slow evaporation of its ethanolic solution in the presence of a few drops of water, at room temperature.

### Refinement

The C- and N- bound hydrogen atoms were positioned with idealized geometry using a riding model with C–H = 0.93 Å or 0.96 Å and N–H = 0.86 Å. The coordinates of the water hydrogen atom were refined. The *U*_iso_(H) values were set at 1.2*U*_eq_(C aromatic, N, O) and 1.5*U*_eq_(C_methyl_). The C14-methyl group exhibits orientational disorder in the positions of H atoms. The two sets of methyl hydrogen atoms were refined with occupancies of 0.52 (9)) and 0.48 (9).

### Crystal data

**Table:**

C_14_H_12_ClNO·0.5H_2_O	*F*(000) = 1064
*M**_r_* = 254.71	*D*_x_ = 1.349 Mg m^−^^3^
Monoclinic, *C*2/*c*	Mo *K*α radiation, λ = 0.71073 Å
Hall symbol: -C 2yc	Cell parameters from 9097 reflections
*a* = 7.6497 (3) Å	θ = 2.4–29.6°
*b* = 12.6829 (5) Å	µ = 0.29 mm^−^^1^
*c* = 25.9694 (10) Å	*T* = 295 K
β = 95.365 (3)°	Rod, colourless
*V* = 2508.54 (16) Å^3^	0.52 × 0.16 × 0.06 mm
*Z* = 8	

### Data collection

**Table:**

Oxford Diffraction Xcalibur diffractometer with a Ruby Gemini detector	2277 independent reflections
graphite	1894 reflections with *I* > 2σ(*I*)
Detector resolution: 10.434 pixels mm^-1^	*R*_int_ = 0.029
ω scans	θ_max_ = 25.3°, θ_min_ = 3.1°
Absorption correction: analytical (*CrysAlis PRO*; Oxford Diffraction, 2009)	*h* = −9→9
*T*_min_ = 0.860, *T*_max_ = 0.984	*k* = −15→15
19774 measured reflections	*l* = −31→31

### Refinement

**Table:**

Refinement on *F*^2^	Primary atom site location: structure-invariant direct methods
Least-squares matrix: full	Secondary atom site location: difference Fourier map
*R*[*F*^2^ > 2σ(*F*^2^)] = 0.041	Hydrogen site location: inferred from neighbouring sites
*wR*(*F*^2^) = 0.095	H atoms treated by a mixture of independent and constrained refinement
*S* = 1.07	*w* = 1/[σ^2^(*F*_o_^2^) + (0.037*P*)^2^ + 1.9211*P*] where *P* = (*F*_o_^2^ + 2*F*_c_^2^)/3
2277 reflections	(Δ/σ)_max_ = 0.001
164 parameters	Δρ_max_ = 0.16 e Å^−^^3^
2 restraints	Δρ_min_ = −0.20 e Å^−^^3^

### Special details

**Table:**

Geometry. All esds (except the esd in the dihedral angle between two l.s. planes) are estimated using the full covariance matrix. The cell esds are taken into account individually in the estimation of esds in distances, angles and torsion angles; correlations between esds in cell parameters are only used when they are defined by crystal symmetry. An approximate (isotropic) treatment of cell esds is used for estimating esds involving l.s. planes.
Refinement. Refinement of *F*^2^ against ALL reflections. The weighted *R*-factor wR and goodness of fit *S* are based on *F*^2^, conventional *R*-factors *R* are based on *F*, with *F* set to zero for negative *F*^2^. The threshold expression of *F*^2^ > σ(*F*^2^) is used only for calculating *R*-factors(gt) etc. and is not relevant to the choice of reflections for refinement. *R*-factors based on *F*^2^ are statistically about twice as large as those based on *F*, and *R*- factors based on ALL data will be even larger.

### Fractional atomic coordinates and isotropic or equivalent isotropic displacement parameters (Å^2^)

**Table:**

	*x*	*y*	*z*	*U*_iso_*/*U*_eq_	Occ. (<1)
C1	0.2652 (2)	0.58130 (14)	0.27335 (7)	0.0377 (4)	
C2	0.2321 (2)	0.59925 (13)	0.21633 (6)	0.0357 (4)	
C3	0.1655 (2)	0.69364 (13)	0.19574 (6)	0.0369 (4)	
H3	0.1451	0.749	0.2179	0.044*	
C4	0.1286 (2)	0.70707 (14)	0.14252 (7)	0.0384 (4)	
C5	0.1625 (2)	0.62336 (15)	0.11091 (7)	0.0447 (4)	
H5	0.1389	0.6307	0.0753	0.054*	
C6	0.2302 (3)	0.52944 (15)	0.13046 (7)	0.0485 (5)	
H6	0.2535	0.4748	0.1082	0.058*	
C7	0.2633 (2)	0.51674 (14)	0.18334 (7)	0.0435 (4)	
H7	0.3064	0.4529	0.1968	0.052*	
C8	0.3568 (2)	0.67382 (13)	0.35616 (6)	0.0330 (4)	
C9	0.2973 (2)	0.59967 (14)	0.38974 (7)	0.0392 (4)	
H9	0.2362	0.5402	0.3772	0.047*	
C10	0.3311 (2)	0.61650 (15)	0.44221 (7)	0.0450 (4)	
C11	0.4205 (3)	0.70309 (16)	0.46236 (7)	0.0524 (5)	
H11	0.4424	0.7123	0.4979	0.063*	
C12	0.4770 (3)	0.77618 (16)	0.42818 (7)	0.0516 (5)	
H12	0.5371	0.8359	0.4409	0.062*	
C13	0.4461 (2)	0.76231 (14)	0.37559 (7)	0.0407 (4)	
H13	0.4851	0.8123	0.3531	0.049*	
C14	0.0552 (3)	0.80902 (15)	0.12042 (8)	0.0542 (5)	
H14A	0.0834	0.865	0.1446	0.081*	0.52 (9)
H14B	−0.07	0.8032	0.1138	0.081*	0.52 (9)
H14C	0.1053	0.824	0.0887	0.081*	0.52 (9)
H14D	0.1418	0.8436	0.1019	0.081*	0.48 (9)
H14E	0.0242	0.8537	0.148	0.081*	0.48 (9)
H14F	−0.0474	0.7949	0.0972	0.081*	0.48 (9)
N1	0.32473 (17)	0.66596 (11)	0.30168 (5)	0.0362 (3)	
H1N	0.3456	0.7217	0.2845	0.043*	
O1	0.24049 (18)	0.49471 (10)	0.29259 (5)	0.0516 (4)	
O2W	0	0.34503 (13)	0.25	0.0413 (4)	
H2W	0.077 (2)	0.3837 (15)	0.2631 (7)	0.05*	
Cl1	0.25269 (9)	0.52457 (5)	0.48445 (2)	0.0763 (2)	

### Atomic displacement parameters (Å^2^)

**Table:**

	*U*^11^	*U*^22^	*U*^33^	*U*^12^	*U*^13^	*U*^23^
C1	0.0362 (9)	0.0364 (10)	0.0401 (9)	−0.0005 (7)	0.0012 (7)	−0.0016 (8)
C2	0.0340 (9)	0.0362 (10)	0.0368 (9)	−0.0055 (7)	0.0030 (7)	−0.0004 (7)
C3	0.0350 (9)	0.0368 (9)	0.0389 (10)	−0.0028 (7)	0.0028 (7)	−0.0064 (8)
C4	0.0356 (9)	0.0388 (10)	0.0401 (10)	−0.0050 (7)	−0.0005 (7)	−0.0018 (8)
C5	0.0503 (10)	0.0495 (11)	0.0337 (9)	−0.0073 (9)	0.0010 (8)	−0.0041 (9)
C6	0.0611 (12)	0.0412 (11)	0.0438 (11)	−0.0018 (9)	0.0090 (9)	−0.0111 (9)
C7	0.0514 (10)	0.0354 (10)	0.0440 (10)	−0.0002 (8)	0.0057 (8)	−0.0023 (8)
C8	0.0322 (8)	0.0330 (9)	0.0339 (9)	0.0055 (7)	0.0028 (7)	−0.0017 (7)
C9	0.0442 (10)	0.0344 (9)	0.0389 (10)	−0.0013 (8)	0.0035 (8)	−0.0021 (8)
C10	0.0534 (11)	0.0441 (11)	0.0381 (10)	0.0026 (9)	0.0077 (8)	0.0044 (8)
C11	0.0641 (12)	0.0601 (13)	0.0321 (10)	−0.0029 (10)	−0.0007 (9)	−0.0071 (9)
C12	0.0566 (12)	0.0477 (11)	0.0497 (11)	−0.0101 (9)	0.0014 (9)	−0.0113 (9)
C13	0.0456 (10)	0.0380 (10)	0.0388 (10)	−0.0027 (8)	0.0051 (8)	−0.0012 (8)
C14	0.0607 (12)	0.0472 (12)	0.0529 (12)	0.0029 (10)	−0.0041 (9)	0.0026 (9)
N1	0.0435 (8)	0.0319 (7)	0.0331 (8)	−0.0034 (6)	0.0025 (6)	0.0023 (6)
O1	0.0757 (9)	0.0354 (7)	0.0417 (7)	−0.0116 (6)	−0.0055 (6)	0.0036 (6)
O2W	0.0476 (10)	0.0314 (10)	0.0442 (10)	0	−0.0003 (8)	0
Cl1	0.1181 (5)	0.0692 (4)	0.0436 (3)	−0.0204 (3)	0.0181 (3)	0.0103 (3)

### Geometric parameters (Å, °)

**Table:**

C1—O1	1.228 (2)	C9—C10	1.380 (2)
C1—N1	1.356 (2)	C9—H9	0.93
C1—C2	1.496 (2)	C10—C11	1.371 (3)
C2—C7	1.387 (2)	C10—Cl1	1.7451 (19)
C2—C3	1.389 (2)	C11—C12	1.380 (3)
C3—C4	1.395 (2)	C11—H11	0.93
C3—H3	0.93	C12—C13	1.375 (2)
C4—C5	1.382 (2)	C12—H12	0.93
C4—C14	1.502 (3)	C13—H13	0.93
C5—C6	1.377 (3)	C14—H14A	0.96
C5—H5	0.93	C14—H14B	0.96
C6—C7	1.382 (3)	C14—H14C	0.96
C6—H6	0.93	C14—H14D	0.96
C7—H7	0.93	C14—H14E	0.96
C8—C13	1.384 (2)	C14—H14F	0.96
C8—C9	1.388 (2)	N1—H1N	0.86
C8—N1	1.417 (2)	O2W—H2W	0.813 (18)
			
O1—C1—N1	122.95 (16)	C8—C9—H9	120.9
O1—C1—C2	121.35 (15)	C11—C10—C9	122.79 (17)
N1—C1—C2	115.69 (15)	C11—C10—Cl1	118.93 (14)
C7—C2—C3	119.37 (16)	C9—C10—Cl1	118.27 (14)
C7—C2—C1	118.28 (15)	C10—C11—C12	117.86 (17)
C3—C2—C1	122.30 (15)	C10—C11—H11	121.1
C2—C3—C4	121.33 (16)	C12—C11—H11	121.1
C2—C3—H3	119.3	C13—C12—C11	121.16 (18)
C4—C3—H3	119.3	C13—C12—H12	119.4
C5—C4—C3	117.58 (16)	C11—C12—H12	119.4
C5—C4—C14	121.24 (16)	C12—C13—C8	119.93 (17)
C3—C4—C14	121.17 (16)	C12—C13—H13	120
C6—C5—C4	122.05 (17)	C8—C13—H13	120
C6—C5—H5	119	C4—C14—H14A	109.5
C4—C5—H5	119	C4—C14—H14B	109.5
C5—C6—C7	119.67 (17)	C4—C14—H14C	109.5
C5—C6—H6	120.2	C4—C14—H14D	109.5
C7—C6—H6	120.2	C4—C14—H14E	109.5
C6—C7—C2	119.98 (17)	H14D—C14—H14E	109.5
C6—C7—H7	120	C4—C14—H14F	109.5
C2—C7—H7	120	H14D—C14—H14F	109.5
C13—C8—C9	119.99 (15)	H14E—C14—H14F	109.5
C13—C8—N1	117.04 (15)	C1—N1—C8	127.99 (14)
C9—C8—N1	122.92 (15)	C1—N1—H1N	116
C10—C9—C8	118.26 (16)	C8—N1—H1N	116
C10—C9—H9	120.9		
			
O1—C1—C2—C7	−33.4 (2)	C13—C8—C9—C10	−0.6 (2)
N1—C1—C2—C7	146.10 (16)	N1—C8—C9—C10	−178.05 (15)
O1—C1—C2—C3	144.07 (17)	C8—C9—C10—C11	0.1 (3)
N1—C1—C2—C3	−36.4 (2)	C8—C9—C10—Cl1	178.80 (13)
C7—C2—C3—C4	0.2 (2)	C9—C10—C11—C12	0.5 (3)
C1—C2—C3—C4	−177.18 (15)	Cl1—C10—C11—C12	−178.23 (15)
C2—C3—C4—C5	−0.7 (2)	C10—C11—C12—C13	−0.5 (3)
C2—C3—C4—C14	179.53 (16)	C11—C12—C13—C8	0.0 (3)
C3—C4—C5—C6	0.1 (3)	C9—C8—C13—C12	0.6 (3)
C14—C4—C5—C6	179.83 (17)	N1—C8—C13—C12	178.17 (16)
C4—C5—C6—C7	1.0 (3)	O1—C1—N1—C8	−4.7 (3)
C5—C6—C7—C2	−1.5 (3)	C2—C1—N1—C8	175.85 (14)
C3—C2—C7—C6	0.9 (3)	C13—C8—N1—C1	169.04 (16)
C1—C2—C7—C6	178.40 (16)	C9—C8—N1—C1	−13.4 (3)

### Hydrogen-bond geometry (Å, °)

**Table:**

*D*—H···*A*	*D*—H	H···*A*	*D*···*A*	*D*—H···*A*
N1—H1N···O2W^i^	0.86	2.2	3.0155 (19)	158
O2W—H2W···O1	0.813 (18)	1.991 (18)	2.7984 (17)	171.9 (19)

Symmetry codes: (i) *x*+1/2, *y*+1/2, *z*.
